# The Regulatory Subunit of Protein Kinase A (Bcy1) in *Candida albicans* Plays Critical Roles in Filamentation and White-Opaque Switching but Is Not Essential for Cell Growth

**DOI:** 10.3389/fmicb.2016.02127

**Published:** 2017-01-05

**Authors:** Xuefen Ding, Chengjun Cao, Qiushi Zheng, Guanghua Huang

**Affiliations:** ^1^State Key Laboratory of Mycology, Institute of Microbiology, Chinese Academy of SciencesBeijing, China; ^2^College of Life Sciences, University of Chinese Academy of SciencesBeijing, China

**Keywords:** *Candida albicans*, PKA regulatory subunit, *Bcy1*, filamentation, white-opaque switching, cAMP signaling pathway

## Abstract

The conserved cAMP-dependent protein kinase (PKA) is composed of the regulatory and catalytic subunits and acts as the central component of the cAMP signaling pathway. In the human fungal pathogen *Candida albicans*, the PKA regulatory subunit Bcy1 plays a critical role in the regulation of cell differentiation and death. It has long been considered that Bcy1 is essential for cell viability in *C. albicans*. In the current study, surprisingly, we found that Bcy1 is not required for cell growth, and we successfully generated a *bcy1/bcy1* null mutant in *C. albicans*. Deletion of *BCY1* leads to multiple cellular morphologies and promotes the development of filaments. Filamentous and smooth colonies are two typical morphological types of the *bcy1/bcy1* mutant, which can undergo spontaneous switching between the two types. Cells of filamentous colonies grow better on a number of different culture media and have a higher survival rate than cells of smooth colonies. In addition, deletion of *BCY1* significantly increased the frequency of white-to-opaque switching on N-acetylglucosamine (GlcNAc)-containing medium. The *bcy1/bcy1* null mutant generated herein provides the field a new resource to study the biological functions of the cAMP signaling pathway in *C. albicans*.

## Introduction

The cAMP signaling pathway regulates a plethora of biological processes in eukaryotic organisms (Wang and Heitman, 1999; Pan et al., 2000; Gancedo, 2001; Chin et al., 2002; Chiaradonna et al., 2008). In the human fungal pathogen *Candida albicans*, this pathway plays a central role in the regulation of morphological transitions, carbon source utilization, quorum sensing, cell death, and virulence (Leberer et al., 2001; Rocha et al., 2001; Phillips et al., 2006; Biswas et al., 2007; Huang et al., 2010; Huang, 2012; Du et al., 2015). In response to environmental stimulation [such as increased CO_2_ levels, N-acetylglucosamine (GlcNAc), serum, and elevated temperatures], cells of *C. albicans* activate the cAMP signaling pathway and undergo morphological changes (Leberer et al., 2001; Phillips et al., 2006; Biswas et al., 2007; Huang et al., 2010; Huang, 2012).

Morphological plasticity is a striking feature of pathogenic *Candida* species and is tightly linked to virulence (Biswas et al., 2007; Whiteway and Bachewich, 2007; Huang, 2012). *C. albicans* can exist in a number of morphological forms, such as the yeast form, filaments (hyphae and pseudohyphae), and white, gray, and opaque cell types (Biswas et al., 2007; Whiteway and Bachewich, 2007; Huang, 2012). Different cell types of *C. albicans* play distinct roles in its life cycle. For example, the yeast cell can be easily disseminated to different tissues through the host circulatory system, while filamentous cells are better at invading tissue and initiating infections (Gow et al., 2012). White cells are more virulent in systemic infections, while opaque and gray cells have an enhanced ability to colonize cutaneous tissues (Tao et al., 2014b). The cAMP-PKA pathway is the major pathway involved in the regulation of morphological transitions and virulence in *C. albicans* (Biswas et al., 2007; Huang, 2012). Ras1 is upstream of the cAMP signaling pathway and is required for serum-induced true hyphal formation, but it is not essential for cell growth and the development of pseudohyphae in *C. albicans* (Feng et al., 1999). In response to extracellular stimuli, the activated Ras protein (Ras1) signals the adenylyl cyclase Cyr1 (also named Cdc35) to increase the synthesis of cAMP in *C. albicans* (Feng et al., 1999; Rocha et al., 2001). Deletion of *CYR1* in *C. albicans* results in slow cell growth and serious defects in filamentation (Rocha et al., 2001). Cyr1 functions as a sensor for multiple extracellular signals including CO_2_, quorum sensing molecules, GlcNAc, and bacterial peptidoglycan (Wang, 2013). The alterations of cAMP levels modulate the activity of the cAMP-dependent protein kinase (PKA).

The PKA kinase is composed of two catalytic and two regulatory subunits in *C. albicans* (Biswas et al., 2007). Tpk1 and Tpk2 are two isoforms of the PKA catalytic subunit, which physically interacts with and is regulated by Bcy1, the regulatory subunit in *C. albicans* (Bockmühl et al., 2001; Cassola et al., 2004; Giacometti et al., 2012; Schaekel et al., 2013). The binding of cAMP to the regulatory subunit leads to the release and activation of the catalytic subunits.Tpk1 and Tpk2 play distinct and redundant roles in the regulation of filamentation, the stress response, and glycogen storage (Bockmühl et al., 2001; Giacometti et al., 2009). Bockmühl et al. (2001) have shown that Tpk1 is required for the formation of filaments on solid media, while Tpk2 is required for filamentation in liquid media. The authors further conclude that the two isoforms of the catalytic subunit are essential for cell viability because they were unable to generate the *tpk1/tpk1 tpk2/tpk2* double mutant (Bockmühl et al., 2001; Giacometti et al., 2009). The PKA regulatory subunit Bcy1 plays a negative role in the regulation of the cAMP signaling pathway in fungal species (Cassola et al., 2004; Giacometti et al., 2006, 2012; Schaekel et al., 2013). Cassola et al. (2004) demonstrated that it is not possible to generate a *BCY1* null mutant in a WT strain of *C. albicans*, since inactivation of *BCY1* leads to constitutive activation of the cAMP/PKA pathway (Cassola et al., 2004). Alternatively, they generated a *bcy1/bcy1 tpk2/tpk2* double mutant by deletion of *BCY1* in a *tpk2/tpk2* background strain. This double mutant exhibits a defect in the development of filaments in response to GlcNAc and serum (Cassola et al., 2004). And deletion of BCY1 affects the nuclear localization of Tpk1, suggesting that Bcy1 may also regulate the activity of the catalytic subunit by controlling its subcellular localization.

In the present study, surprisingly, we found that Bcy1 is not essential for cell growth of *C. albicans*. We successfully deleted both alleles of *BCY1* and generated a *bcy1/bcy1* null mutant in a laboratory wild type strain of *C. albicans*. This mutant provides an opportunity to revisit the biological roles of the PKA regulatory subunit in this important fungal pathogen. Deletion of *BCY1* in *C. albicans* leads to multiple cellular morphologies and hyperfilamentation in certain media. We further show that Bcy1 plays an important role in the regulation of carbon source utilization and in white-opaque switching.

## Materials and methods

### Culture conditions, strains, and plasmids

The strains used in this study are listed in Table S1. YPD medium (2% glucose, 2% peptone, 1% yeast extract) and modified Lee's glucose medium (Huang et al., 2010) were used for routine culture of *C. albicans*. The red dye phloxine B (5 μg/mL) was added to the solid medium for the filamentation and white-opaque switching assays. Media used for spot serial dilution growth assays: YPD, Lee's (Lee's medium without sugar) (Lee et al., 1975), Lee's media with different carbon sources (replacement of glucose with 1.25% fructose or 3% ethanol plus 2% glycerol), YNB media [yeast nitrogen base with 0.5% ammonium sulfate and 3 amino acids (0.13 g/L leucine, 0.03 g/L histidine, and 0.04 g/L arginine) and different carbon sources (2% glucose, 2% fructose, 2% mannitol, or 4% glycerol)].

*BCY1* were deleted in two WT strains (SN152 and SN152a) using the same strategy as described below. The two alleles of *BCY1* were deleted using the fusion PCR strategy (Noble and Johnson, 2005). The first allele of *BCY1* was replaced with the fusion PCR products of the *CdHIS1* marker amplified from plasmid pSN52. The second allele of *BCY1* was deleted with the fusion PCR products of the *CmLEU2* marker amplified from pSN40. The primers used for the PCR reactions are listed in Table S2. To construct the *BCY1*-reconstituted strain, the fusion PCR product of three fragments (the *CdARG4* marker amplified from plasmid pSN69, and fragments of *BCY1* 3′-UTR and *BCY1* ORF plus 5′-UTR) was used for transformation of the *bcy1/bcy1* mutant. The two *BCY1*-related fragments were amplified from genomic DNA of *C. albicans* (SC5314) with primer pairs (BCY1-5F-COM plus BCY1-5R-COM and BCY1-3R-COM plus BCY1-3R-COM, respectively).

Plasmid pACT1 was used to construct the *TPK1* and *TPK2-*overexpressing plasmids (Huang et al., 2010). The PCR products of *TPK1* were digested with *Eco*RV and *Hind*III and inserted into the *Eco*RV/*Hind*III site of pACT1 to generate plasmid pACT-TPK1. The PCR products of *TPK2* were digested with *Stu*I and *Hind*III and inserted into the *Eco*RI/*Hind*III site of pACT1, to generate plasmid pACT-TPK2. The AscI-linearized overexpressing plasmids were used for transformation of the WT and *bcy1/bcy1* mutant.

### White-opaque switching assay

White-opaque switching assays were performed as described previously (Xie et al., 2013). Lee's glucose and Lee's GlcNAc media were used for the quantitative switching assays. The cells were cultured on the plates at 25°C for 5 days. The cell identity was assessed by cellular morphology and verified by cell type-specific genes (data not shown).

### Filamentation assays

Lee's glucose, Lee's GlcNAc, and YPD media were used for the filamentation assays. The cells were cultured at 25° and 37°C as indicated in the figure legends. For quantitative filamentous-smooth colony type switching assays (**Figure 4**), cells from a filamentous or smooth colony grown on Lee's glucose medium for 3 days were resuspended and cultured in liquid Lee's glucose for 24–96 h at 25°C. A small aliquot of cells was collected at the time point indicated and replated on YPD plates. After 5 days of growth at 30°C, colonies of the smooth and filamentous types were counted. The switching frequency = (number of colonies with alternative phenotype/total colony number) × 100%.

### PI and DAPI staining assays

The cells were grown in liquid Lee's glucose medium for 48 h at 25°C and collected for propidium iodide (PI) and 4′-6-diamidino-2-phenylindole (DAPI) staining assays as described previously (Du et al., 2015). The cells were washed with 1 × phosphate-buffered saline (PBS) and resuspended in 1 × PBS. PI was added to the cells at a concentration of 2 μg/mL. The cells were stained for 15 min at room temperature in the dark with slight shaking and used for microscopy assays. For the DAPI staining assays, cells collected from liquid Lee's glucose medium were first fixed in 70% ethanol for 20 min and then stained with 1 μg/mL of DAPI. The cells were then washed with 1 × PBS and used for microscopy assays.

### Quantitative real-time PCR (qRT-PCR) assay

Cells were grown Lee's GlcNAc plates at 25°C for 3 days and collected for total RNA extraction. The qRT-PCR assay was performed as described in our previous report (Tao et al., 2014a). Total RNA was used to synthesize cDNA with RevertAid H Minus reverse transcriptase (Thermo Scientific). Quantification of transcripts was performed in Bio-Rad CFX96 real-time PCR detection system using SYBR green. The relative expression level of each gene was normalized to that of *C. albicans ACT1*.

## Results

### Generation of the *bcy1/bcy1* null mutant in *C. albicans*

**Figure 1:**
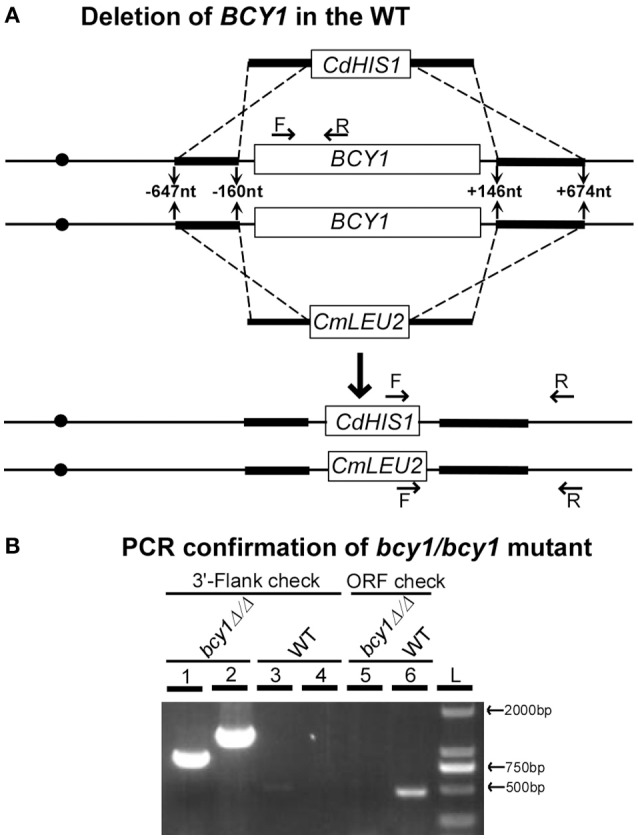
**Deletion of both alleles of ***BCY1*** in ***C. albicans***. (A)** Strategy for *BCY1* deletion. Fusion PCR deletion assays were used to delete the two alleles of *BCY1* as described in the Methods Section. *CdHIS1* and *CmLEU2* were used as selective markers for *BCY1* deletion. **(B)** PCR confirmation of *BCY1* deletion in the WT strain SN152. The primers used are indicated in **(A)**. Lanes 1 and 3: 3′-flanking checking primers for *CdHIS1* integration; lanes 2 and 4: 3′-flanking checking primers for *CmLUE2* integration; lanes 5 and 6: *BCY1* ORF checking primers. L, DNA ladder.

Although the PKA regulatory subunit plays critical roles in a variety of biological processes in fungal species, the gene encoding this subunit is not essential for cell growth in many fungi including the model organisms, *Saccharomyces cerevisiae* (Cannon and Tatchell, 1987; Toda et al., 1987), *Schizosaccharomyces pombe* (DeVoti et al., 1991), and *Neurospora crassa* (Bruno et al., 1996), and the human fungal pathogens, *Aspergillus fumigates* (Zhao et al., 2006) and *Cryptococcus neoformans* (D'Souza et al., 2001). However, it has been thought that *BCY1*, the sole gene encoding the PKA regulatory subunit in *C. albicans*, is an essential gene (Cassola et al., 2004). Considering the conserved feature of the cAMP signaling pathway, we suspected that *BCY1* might not be an essential gene in *C. albicans*, and thus the failure to obtain its null mutant in a previous report (Cassola et al., 2004) could be due to technical reasons. Using a fusion PCR deletion and prototrophic selection strategy (Noble and Johnson, 2005), we successfully deleted the two alleles of *BCY1* in a WT strain of *C. albicans* (SN152, Figure 1). Correct integration of the *CdHIS1* and *CmLEU2* markers into the *BCY1* locus was confirmed using PCR with two sets of flanking checking primers (Figure 1B, lanes 1–4). Moreover, one set of ORF primers was used to verify the absence of the *BCY1* ORF region in the genome (Figure 1B, lanes 5 and 6). These results indicate that the two alleles of *BCY1* were successfully deleted and replaced by the *CdHIS1* and *CmLEU2* markers, respectively. Therefore, as in other previously described fungi (Cannon and Tatchell, 1987; Toda et al., 1987; DeVoti et al., 1991; Bruno et al., 1996; D'Souza et al., 2001 and Zhao et al., 2006), the conserved PKA regulatory subunit Bcy1 is also not required for cell viability in *C. albicans*.

### Multiple morphologies of the *bcy1/bcy1* null mutant

**Figure 2:**
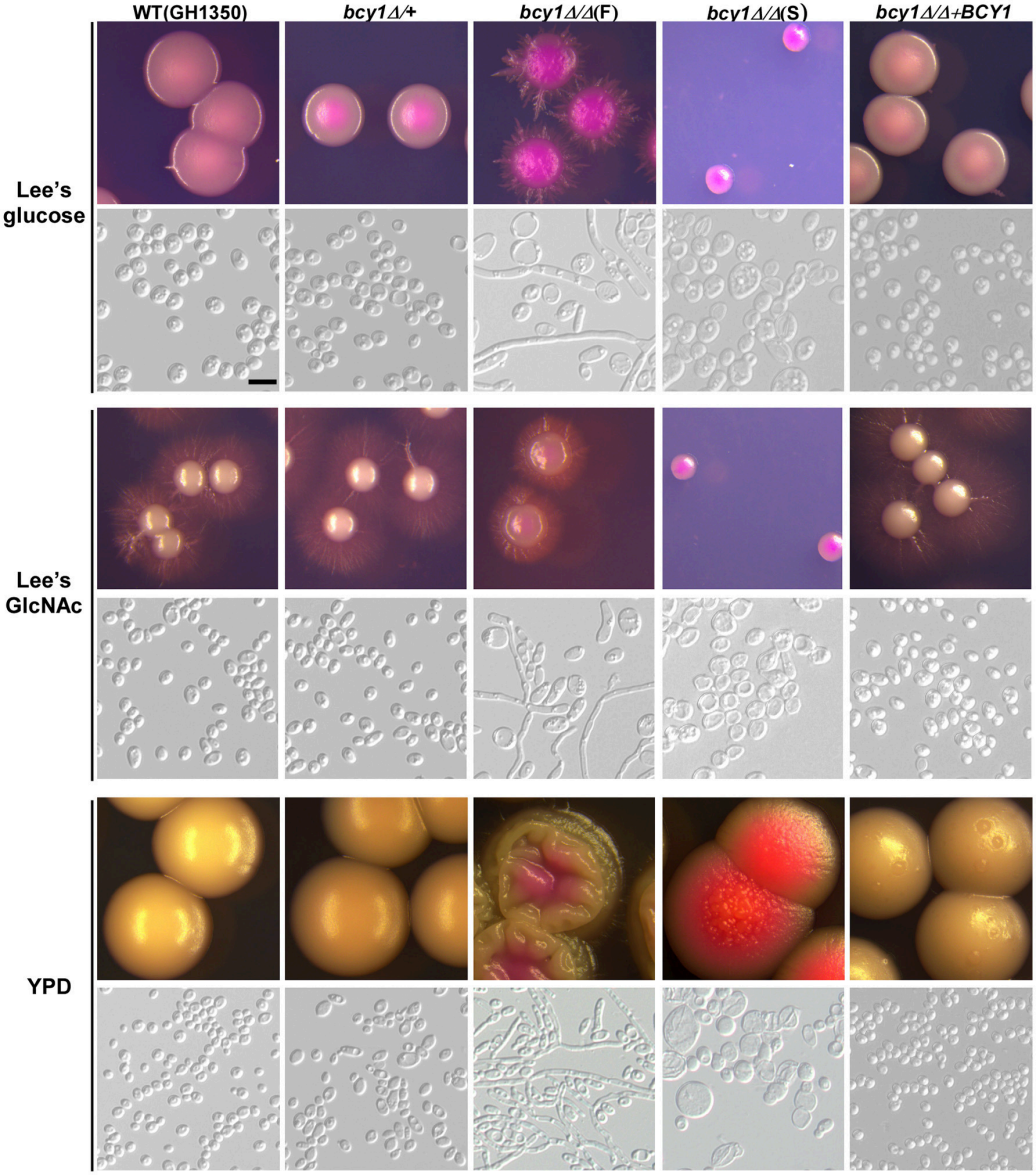
**Cellular and colony morphologies of the WT, ***BCY1/bcy1,*** and ***bcy1/bcy1*** mutants, and the BCY1-reconstituted strain on Lee's glucose, Lee's GlcNAc, and YPD media at 25°C**. The cells were cultured on Lee's glucose and Lee's GlcNAc medium plates for 5 days or on YPD plates for 3 days. The *bcy1/bcy1* mutant consists of two colony phenotypes: smooth (S) and filamentous (F). Scale bar, 10 μm.

**Figure 3:**
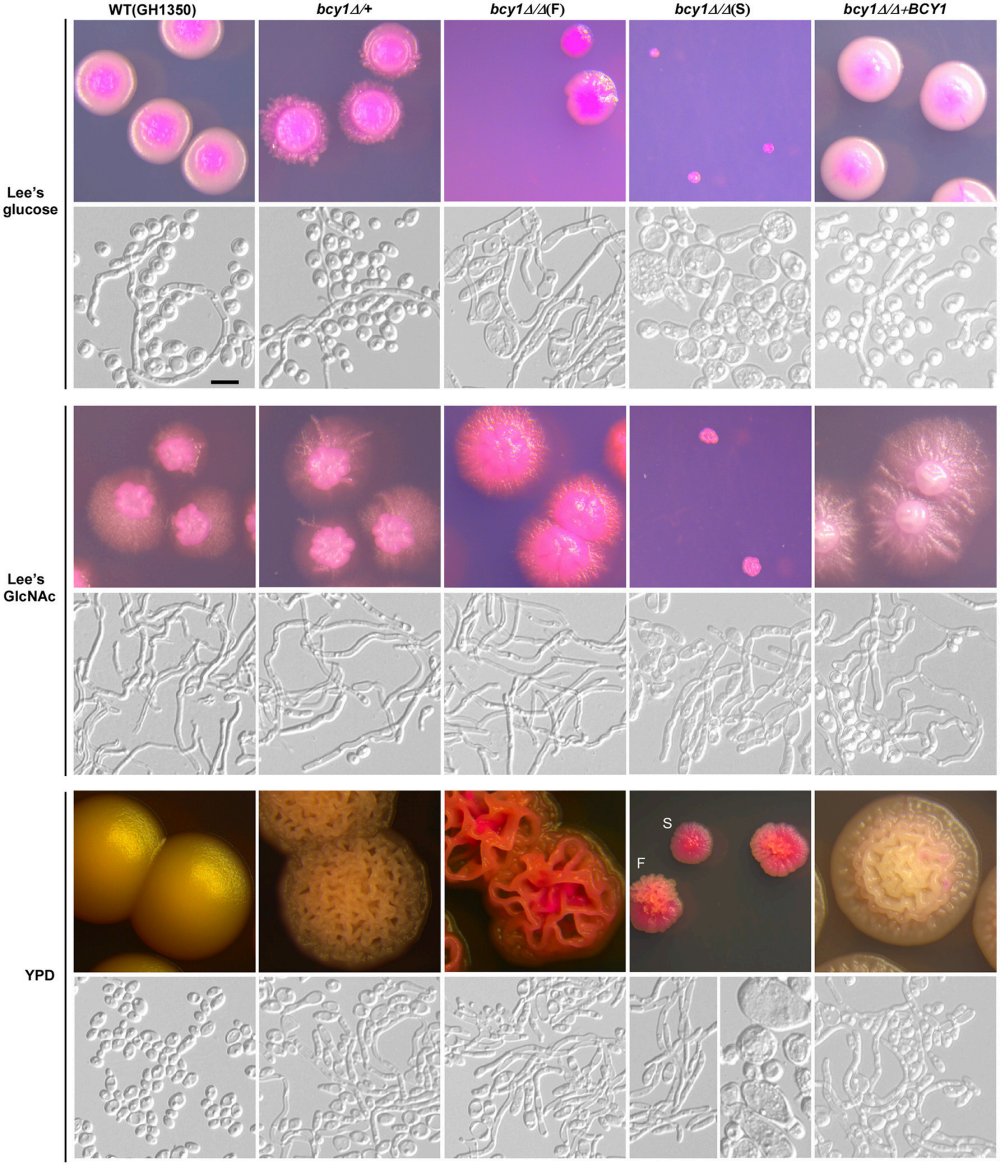
**Cellular and colony morphologies of the WT, ***BCY1/bcy1***, and ***bcy1/bcy1*** mutants, and BCY1-reconstituted strain on Lee's glucose, Lee's GlcNAc, and YPD media at 37°C**. The cells were cultured on Lee's glucose and Lee's GlcNAc medium plates for 5 days or on YPD plates for 3 days. At 37°C, all colonies of the *bcy1/bcy1* mutant underwent hyper-filamentation on Lee's GlcNAc medium. Scale bar, 10 μm.

To evaluate the function of Bcy1 during filamentous development of *C. albicans*, we cultured the cells of WT, *BCY1/bcy1, bcy1/bcy1*, and BCY1-reconstituted strains on three different media (Lee's glucose, Lee's GlcNAc, and YPD) at two temperatures (25 and 37°C). These culture conditions were used because the three media exhibit different levels of filamentation induction in *C. albicans*. A high temperature (37°C) promotes filamentation, whereas a low temperature (25°C) favors yeast cell growth. Therefore, a combination of these conditions would facilitate the discrimination of the filamentation ability of different strains. At 25°C (Figure 2), deletion of one allele of *BCY1* had no obvious effect on filamentous growth. Consistently, the BCY1-reconstituted strain also exhibited a similar phenotype to that of the WT control. However, the *bcy1/bcy1* null mutant displayed a serious growth defect and had two distinct colony phenotypes (smooth and filamentous) on all three media. Cells of the smooth colonies were swollen and looked unhealthy, whereas filamentous cells had a much healthier appearance. On Lee's GlcNAc medium, a portion of the *bcy1/bcy1* mutant cells exhibited an opaque-like phenotype (Figure 2). At 37°C (Figure 3), two colony types of the *bcy1/bcy1* mutant were observed on three media. On Lee's glucose medium, filamentous colonies of the *bcy1/bcy1* mutant underwent more robust filamentation than the WT, *BCY1/bcy1*, and BCY1-reconstituted strains, whereas on Lee's GlcNAc medium, all four strains exhibited strong filamentation at 37°C. These results are consistent with previous reports showing that GlcNAc is a potent yeast-to-filamentous growth inducer (Simonetti et al., 1974). The *bcy1/bcy1* mutant showed the most robust filametation on YPD medium, while the *BCY1/bcy1* and BCY1-reconstituted strains exhibited an intermediate level of filamentation at 37°C. The WT control maintained the yeast form on YPD medium at 37°C. These results suggest that Bcy1 plays a critical role in the regulation of filamentation and that the dosage of Bcy1 also affects this biological process.

### The *bcy1/bcy1* null mutant can switch between the smooth and filamentous phenotypes

**Figure 4:**
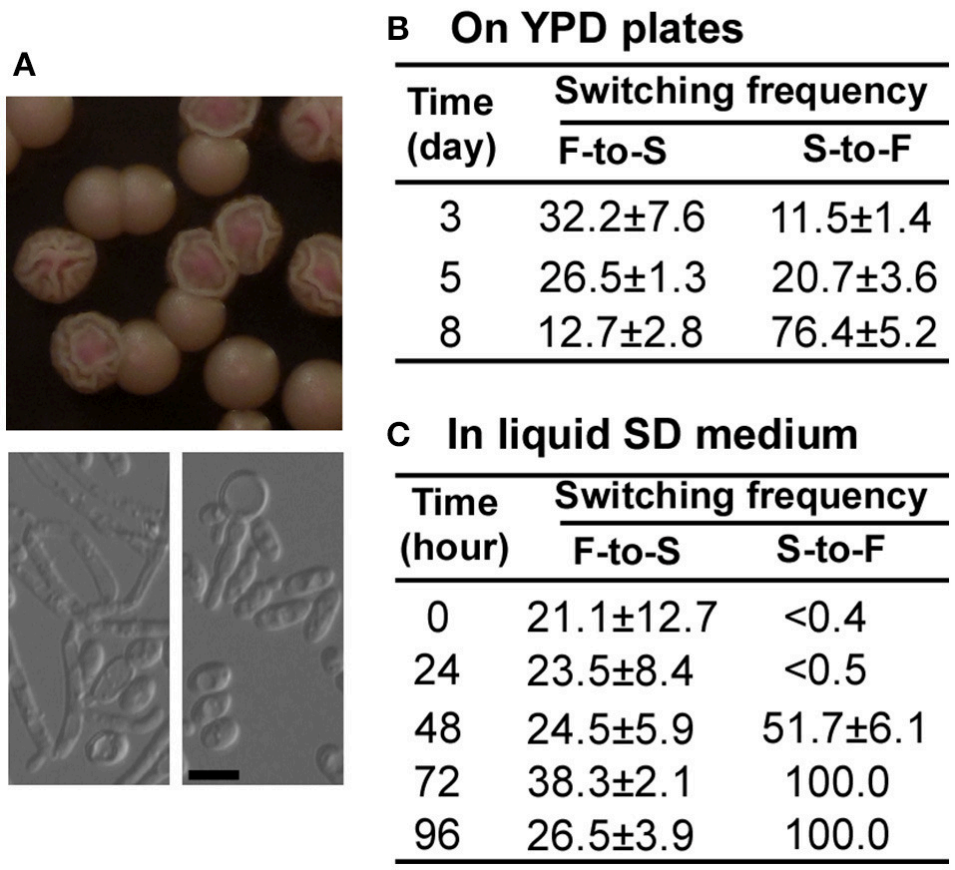
**Switching between smooth (S) and filamentous (F) forms of the ***bcy1/bcy1*** mutant at 30°C. (A)** Cellular and colony morphologies of the *bcy1/bcy1* mutant. The cells were cultured on YPD medium for 4 days. Scale bar, 10 μm. **(B)** Switching frequencies of S-to-F and F-to-S on YPD medium. Smooth and filamentous cells were plated on YPD medium and cultured for 3–8 days. **(C)** Switching frequencies of S-to-F and F-to-S in liquid SD medium. Cells were incubated in liquid SD medium for 0–96 h and then replated on YPD medium plates for 5 days.

The *bcy1/bcy1* mutant has two colony phenotypes: smooth and filamentous (Figures 2, 3, 4A). To test whether the two phenotypes could switch between each other, we cultured the two cell types on YPD medium and calculated the switching frequencies of the original to alternative cell type after 3, 5, and 8 days at 30°C (Figure 4B). Extension of incubation on solid medium promoted the filamentous phenotype. To further characterize the switching feature of the *bcy1/bcy1* mutant, the smooth and filamentous colonies were cultured in liquid Lee's glucose medium for 0–96 h at 25°C (Figure 4C). The cells were then replated onto YPD medium and cultured at 30°C for 5 days. As shown in Figure 4C, the filamentous-to-smooth (F-to-S) switching frequencies at different time points were similar (20–40%), whereas the smooth-to-filamentous (S-to-F) switching frequencies increased dramatically with extension of the initial culture time.

### Effect of overexpression of *TPK1* and *TPK2* in the *bcy1/bcy1* null mutant

**Figure 5:**
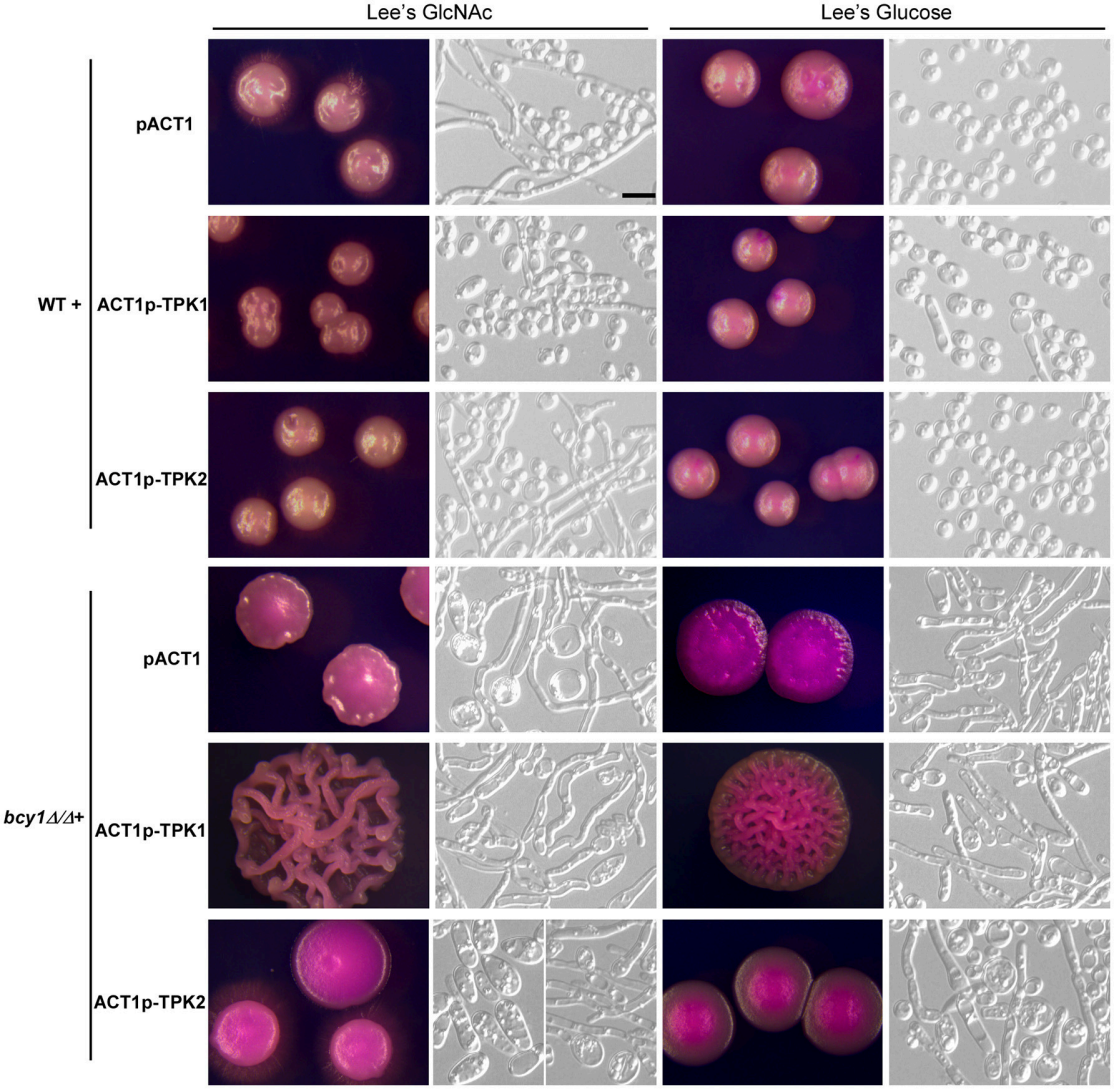
**Overexpression of ***TPK1*** and ***TPK2*** in the WT and ***bcy1/bcy1*** mutant**. Cells were cultured on Lee's glucose and Lee's GlcNAc media at 25°C for 5 days. Scale bar, 10 μm.

Deletion of *BCY1* causes constitutive activation of the PKA kinase. We examined the effect of overexpression of *TPK1* and *TPK2*, which encode the two isoforms of the catalytic subunit, in the *bcy1/bcy1* null mutant. As shown in Figure 5, overexpression of *TPK1* in the *bcy1/bcy1* null mutant resulted in hyper-filamentation at 25°C, while overexpression of *TPK2* did not promote filamentation but led to the formation of two types of colonies: filamentous and opaque-like. On Lee's GlcNAc medium, one colony type was similar to the opaque phenotype, while the other underwent hyper filamentation. Of note, it was very difficult to obtain TPK2-overexpressing transformants in the *bcy1/bcy1* null mutant, suggesting that overexpression of *TPK2* may cause rapid cell death.

### Deletion of *BCY1* promotes cell death

**Figure 6:**
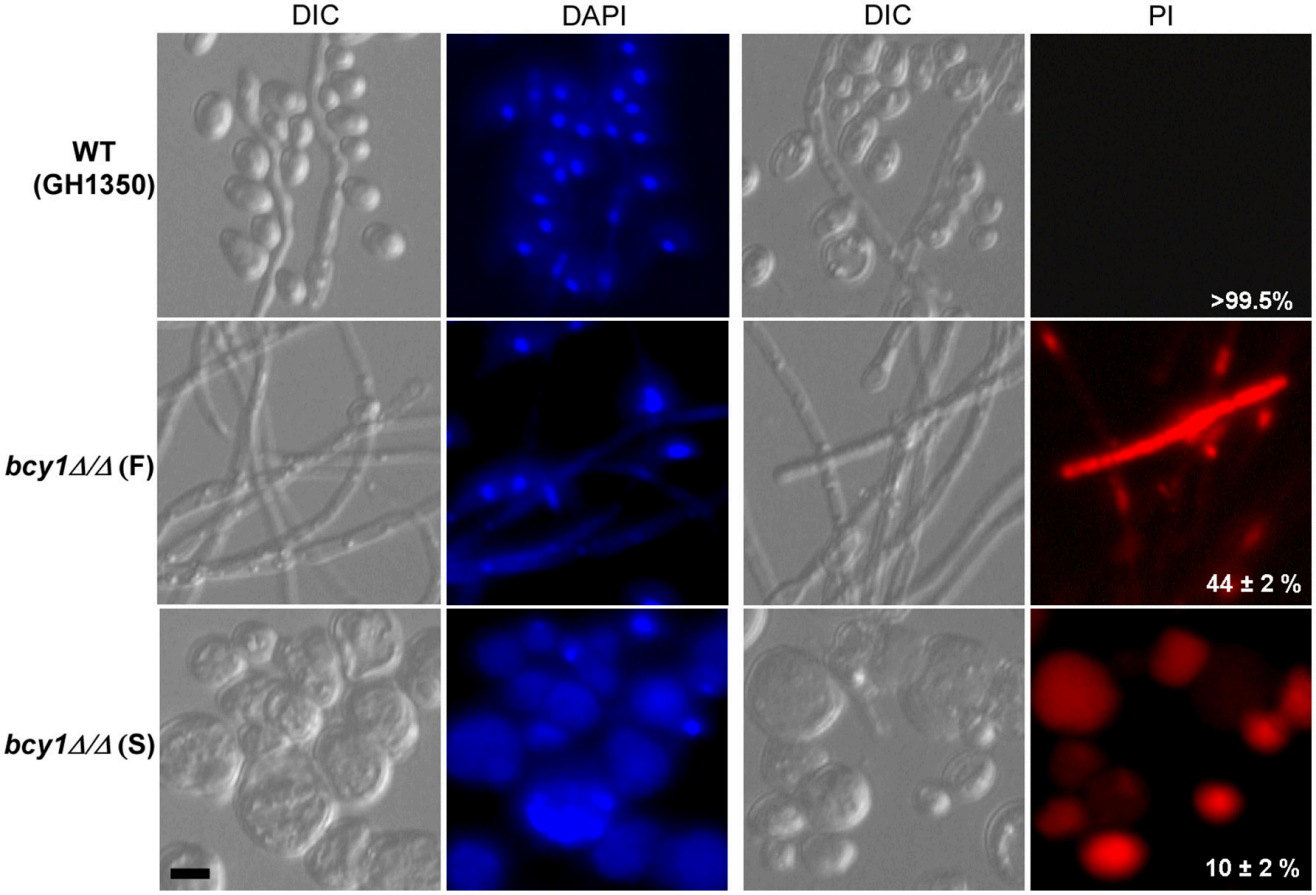
**Deletion of ***BCY1*** in ***C. albicans*** promotes cell death, especially in the ***bcy1/bcy1*** smooth (S) form**. Cells of the WT, *bcy1/bcy1* filamentous (F), and *bcy1/bcy1* smooth (S) forms were incubated in Lee's glucose liquid medium for 48 h with shaking, and then used for DAPI and PI staining assays. The percentages (on PI-images) represent the survival rates using plating assays. DIC, differential interference contrast. Scale bar, 10 μm.

The cAMP signaling pathway regulates cell death in *C. albicans* (Phillips et al., 2006). Because the deletion of *BCY1* results in constitutive activation of this pathway, we next tested the viability of *bcy1/bcy1* mutant cells during incubation in Lee's glucose medium. Cells of the WT, smooth, and filamentous types of the *bcy1/bcy1* null mutant were grown in liquid Lee's glucose medium for 48 h at 25°C. The cells were then collected and stained with DAPI and PI. As shown in Figure 6, cells of the WT and filamentous form of the *bcy1/bcy1* mutant had intact DAPI-stained nuclei, while most cells of the smooth type of the *bcy1/bcy1* mutant had a fragmented nucleus or had no intact nuclei. PI staining verified that most cells of the smooth form of the *bcy1/bcy1* mutant underwent cell death. Quantitative assays demonstrated that more than 95% of the smooth cells and approximately 70% of filamentous cells of the *bcy1/bcy1* mutant were dead after 48 h of incubation in Lee's glucose medium at 25°C. Of note, more than 99% the WT control cells remained viable under the same culture conditions.

### Role of *Bcy1* in the regulation of white-opaque switching

**Figure 7:**
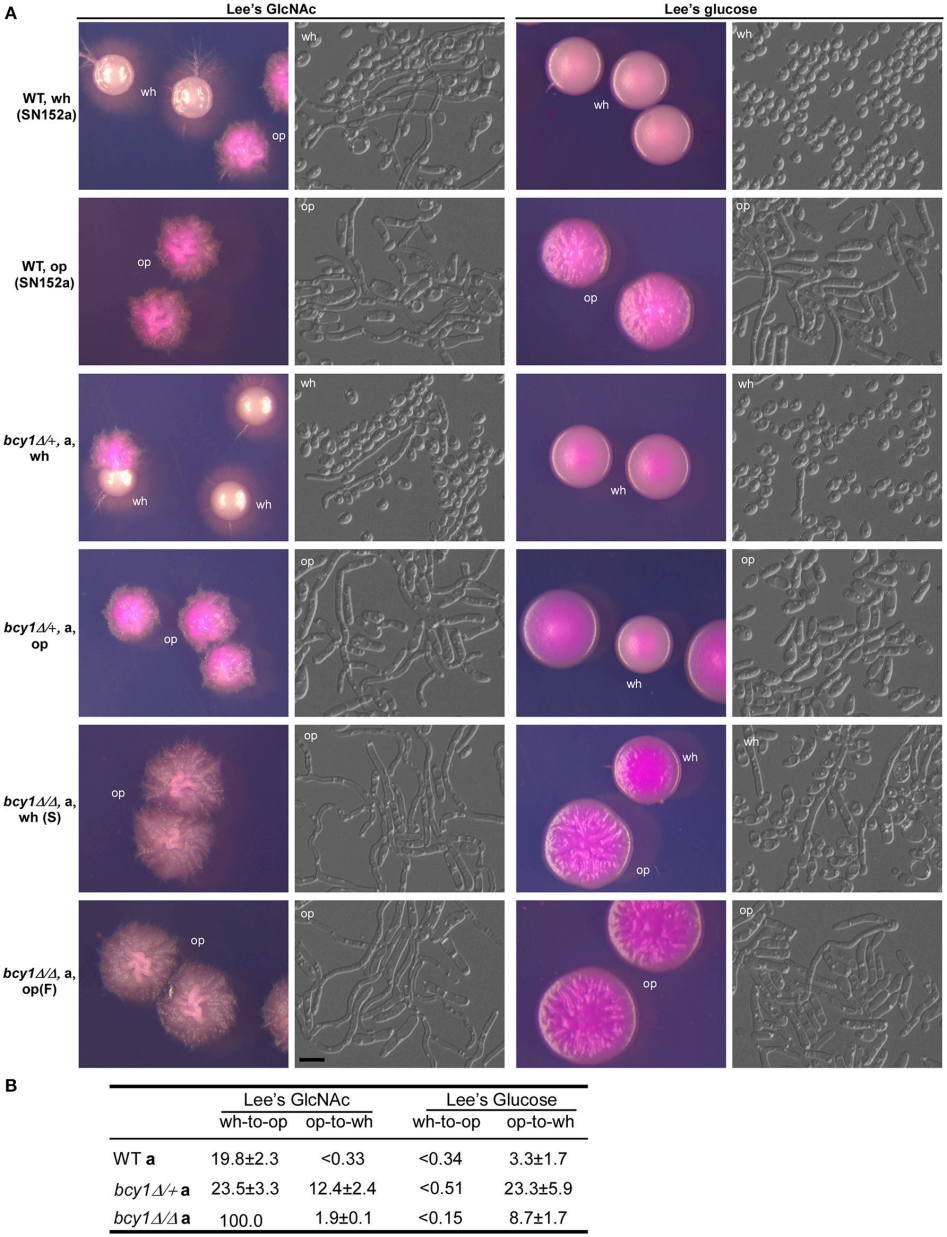
**Deletion of ***BCY1*** affects white-opaque switching in ***C. albicans*****. Cells were cultured on Lee's glucose and Lee's GlcNAc media at 25°C for 5 days. Scale bar, 10 μm. **(A)** Cellular and colony morphologies of the WT, *BCY1/bcy1*, and *bcy1/bcy1* mutants. On Lee's GlcNAc medium, no white colonies were observed since white cells switched to the opaque phenotype under this culture condition. Wh, white; op, opaque. **(B)** White-to-opaque and opaque-to-white switching frequencies of the WT, *BCY1/bcy1*, and *bcy1/bcy1* mutants.

Huang et al. (2010) have demonstrated that activation of the cAMP signaling pathway promotes white-opaque switching in *C. albicans* (Huang et al., 2010). As shown in Figure 2, deletion of *BCY1* in the WT strain (*MTL*a/α) induced the formation of an opaque-like cell type on Lee's GlcNAc medium. Therefore, we deleted *BCY1* in a white-opaque switchable *MTL*a/Δ strain (SN152a Tao et al., 2014a) to generate the *BCY1/bcy1* and *bcy1/bcy1* mutants (*MTL*a/Δ). The colony and cellular morphologies of the WT, *BCY1/bcy1*, and *bcy1/bcy1* mutants (*MTL*a/Δ) are shown in Figure 7A. On both Lee's GlcNAc and Lee's glucose media, the *BCY1/bcy1* mutant exhibited similar colony and cellular phenotypes to those of the WT strain. Both the smooth and filamentous types of the *bcy1/bcy1* mutant could undergo white-opaque switching on Lee's glucose medium. However, both types underwent hyperfilamentation on Lee's GlcNAc medium. Replating and cell type-specific gene expression assays indicated that on Lee's GlcNAc medium, the filamentous cells had an opaque identity (Figure S1). Quantitative switching assays demonstrated that deletion of both alleles of *BCY1* caused a mass conversion to the opaque phenotype on Lee's GlcNAc medium (Figure 7B). These results suggest that Bcy1 plays a negative role in the regulation of the white-to-opaque transition under this culture condition.

### Role of *Bcy1* in the regulation of carbon source utilization

**Figure 8:**
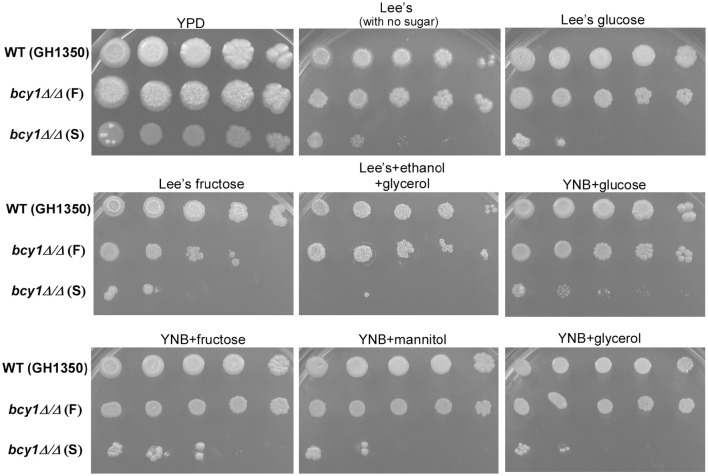
**Bcy1 regulates carbon source utilization**. Cells of the WT, *bcy1/bcy1* filamentous (F), and *bcy1/bcy1* smooth (S) forms were adjusted to 5 × 10^5^ cells/mL, and then spotted onto nine different medium plates at 10-fold serial dilutions. The plates were incubated at 37°C for 4 days. Media used: rich medium YPD; Lee's, Lee's medium containing no sugar; Lee's glucose, Lee's medium containing 1.25% glucose; Lee's fructose, Lee's medium containing 1.25% fructose; Lee's+ethanol+glycerol, Lee's medium containing 3% ethanol and 2% glycerol; YNB+glucose (fructose, mannitol, glycerol), YNB plus 2% glucose (2% fructose, 2% mannitol, or 4% glycerol).

Next, we tested whether the regulatory subunit Bcy1 was involved in the regulation of carbon source utilization in *C. albicans*. As shown in Figure 8, nine media (including rich YPD medium, four Lee's media, and four YNB media containing different types of carbon sources) were used for this assay. The filamentous form of the *bcy1/bcy1* mutant grew well on all media, although its growth rate was slower than that of the WT. The smooth form of the *bcy1/bcy1* mutant grew well on YPD medium. However, the cells of this form showed a serious growth defect on both Lee's and YNB media. This defect did not appear to be related to the fermentative or non-fermentative features of the carbon sources.

## Discussion

The cAMP/PKA pathway plays a critical role in the regulation of a number of features of the human fungal pathogen *C. albicans* (Hogan and Sundstrom, 2009; Huang, 2012). The regulatory subunit Bcy1 has been considered to be essential in this fungus (Cassola et al., 2004). In this study, we demonstrate that the two alleles of *BCY1* could be deleted in *C. albicans*. Given the conserved feature of the cAMP signaling pathway in fungal species, our finding is reasonable because the orthologs of Bcy1 in several fungal species (such as Bcy1 in *S. cerevisiae*, Csg1 in *S. pombe*, and pkaR in *Aspergillus fumigatus* and *Cryptococcus neoformans*) are not essential for cell viability (Cannon and Tatchell, 1987; Toda et al., 1987; DeVoti et al., 1991; Bruno et al., 1996; D'Souza et al., 2001; Zhao et al., 2006). Based on the *bcy1/bcy1* null mutant generated in *C. albicans*, we re-evaluated the roles of Bcy1 in the regulation of filamentation, cell growth, and carbon source utilization. We also found that Bcy1 regulates white-opaque switching in *C. albicans*.

Deletion of *CYR1*, the sole gene encoding the adenylyl cyclase in *C. albicans*, completely blocked filamentation in response to several potent inducers including serum, CO_2_, and bacterial peptidoglycan (Rocha et al., 2001; Klengel et al., 2005; Xu et al., 2008). Activation of the cAMP-PKA pathway by ectopic expression of the activating form of Ras1 (Ras1V13) or deletion of the high affinity cyclic nucleotide phosphodiesterase-encoding gene *PDE2* results in hyperfilamentation in *C. albicans* (Feng et al., 1999; Jung and Stateva, 2003). As expected, deletion of *BCY1* in *C. albicans* promotes filamentation under conditions favoring yeast cell growth (such as at low temperature and in rich media, Figures 2, 3). Similar to the *pde2/pde2* mutant (Jung and Stateva, 2003), the PKA catalytic subunit could be constitutively activated in the *bcy1/bcy1* mutant. Moreover, the phenotypes of the *bcy1/bcy1* mutant are highly similar to the hyperactive *CYR1* mutant (Bai et al., 2011). The activated cAMP-PKA pathway then modulates downstream transcription factors (such as Efg1 and Flo8), which regulate filament-specific gene expression and promote filamentation (Bockmühl and Ernst, 2001; Cao et al., 2006).

Mutation of the PKA regulatory subunit in *S. cerevisiae* causes a variety of phenotypes (Cannon et al., 1990). In *C. albicans*, we found that deletion of *BCY1* also resulted in multiple colony and cellular phenotypes including yeast, filamentous, and opaque-like forms (Figure 2). Interestingly, different cell types of the *bcy1/bcy1* mutant exhibited different cell growth and carbon nutrient utilization abilities (Figure 8). Filamentous cells grew much better than cells of the smooth (yeast) form on all media, suggesting that both fermentative and non-fermentative carbon sources can be utilized by filamentous cells of the mutant. Switching between the filamentous and yeast cell forms can occur (Figure 4). Filamentous cells are healthier and display a better survival rate than cells of the smooth form when grown in regular media. Moreover, an extended culture time (which may represent a stressful condition) appeared to promote yeast-to-filamentous cell growth in the *bcy1/bcy1* mutant. These results suggest that deletion of *BCY1* in *C. albicans* promotes cell death, potentially due to constitutive activation of the cAMP-PKA pathway. Consistent with this idea, Phillips et al. (2006) demonstrated that the protein level of Bcy1 declined significantly during acetic acid-induced programmed cell death (Phillips et al., 2006). Filamentation in the *bcy1/bcy1* mutant could be a strategy of cells to avoid cell death or to improve their anti-stress abilities. Consistent with our study, Laprade et al. (2016) recently reported that filamentation of *C. albicans* provides protection against antifungal-induced programmed cell death (Laprade et al., 2016).

White-opaque switching is another important feature of *C. albicans* and is involved in the regulation of virulence, sexual mating, and stress responses (Slutsky et al., 1987; Lohse and Johnson, 2009; Soll, 2009). Inactivation of the cAMP signaling pathway by the deletion of *CYR1* suppresses GlcNAc and CO_2_-induced white-to-opaque switching, whereas activation of this pathway by ectopic expression of the activating form of *RAS1, RAS1V13*, or deletion of *PDE2* promotes the opaque phenotype (Huang et al., 2009, 2010). Consistent with these observations, deletion of *BCY1* promotes white-to-opaque switching in *C. albicans* (Figure 7). This promoting effect is dosage-dependent because the deletion of one allele of *BCY1* leads to a moderate increase in this switch and deletion of both alleles causes a mass conversion on GlcNAc-containing media. GlcNAc is a potent inducer of the opaque phenotype. Consistent with the phenotype in the *bcy1/bcy1* mutant, Huang et al. (2010) reported that the deletion of *PDE2* also results in a mass white-to-opaque conversion on GlcNAc-containing media (Huang et al., 2010).

In summary, we successfully generated a *bcy1/bcy1* mutant in *C. albicans*, which clarifies the essential role of this PKA regulatory subunit and provides a new avenue to study the cAMP-PKA pathway in this medically important pathogen. Our study also provides new insights into the functional roles of Bcy1 in the regulation of filamentation, carbon source utilization, and white-opaque switching. The results reported herein further confirm the conserved features and central role of the cAMP-PKA pathway in the regulation of a variety of biological features of *C. albicans*.

## Author contributions

XD and GH designed the study. XD, CC, and QZ performed experiments. GH, XD, CC, and QZ analyzed data. GH, XD, CC, and QZ wrote the manuscript.

### Conflict of interest statement

The authors declare that the research was conducted in the absence of any commercial or financial relationships that could be construed as a potential conflict of interest.

## Supplementary material

Figure S1 **Relative gene expression levels in white and opaque cells**. Cells collected from Lee's GlcNAc medium plates (three days, at 25°C) were used for qRT-PCR assays. The values of the expression level of each gene in white cells of the WT strain were set as “1.” White and opaque cells of the WT served as controls. *bcy1/bcy1* op, opaque cells plated on Lee's GlcNAc medium; *bcy1/bcy1* original wh, white cells plated on Lee's GlcNAc medium. *ACT1* served as the reference gene for normalization.

Table S1 **Strains used in this study**.

Table S2 **Primers used in this study**.
