# Supplementary figures and images for: The Regulatory Subunit of Protein Kinase A (Bcy1) in Candida albicans Plays Critical Roles in Filamentation and White-Opaque Switching but Is Not Essential for Cell Growth
Source: Front Microbiol. 2017 Jan 5;7:2127. doi: 10.3389/fmicb.2016.02127 (PMC5215307; doi:10.3389/fmicb.2016.02127)

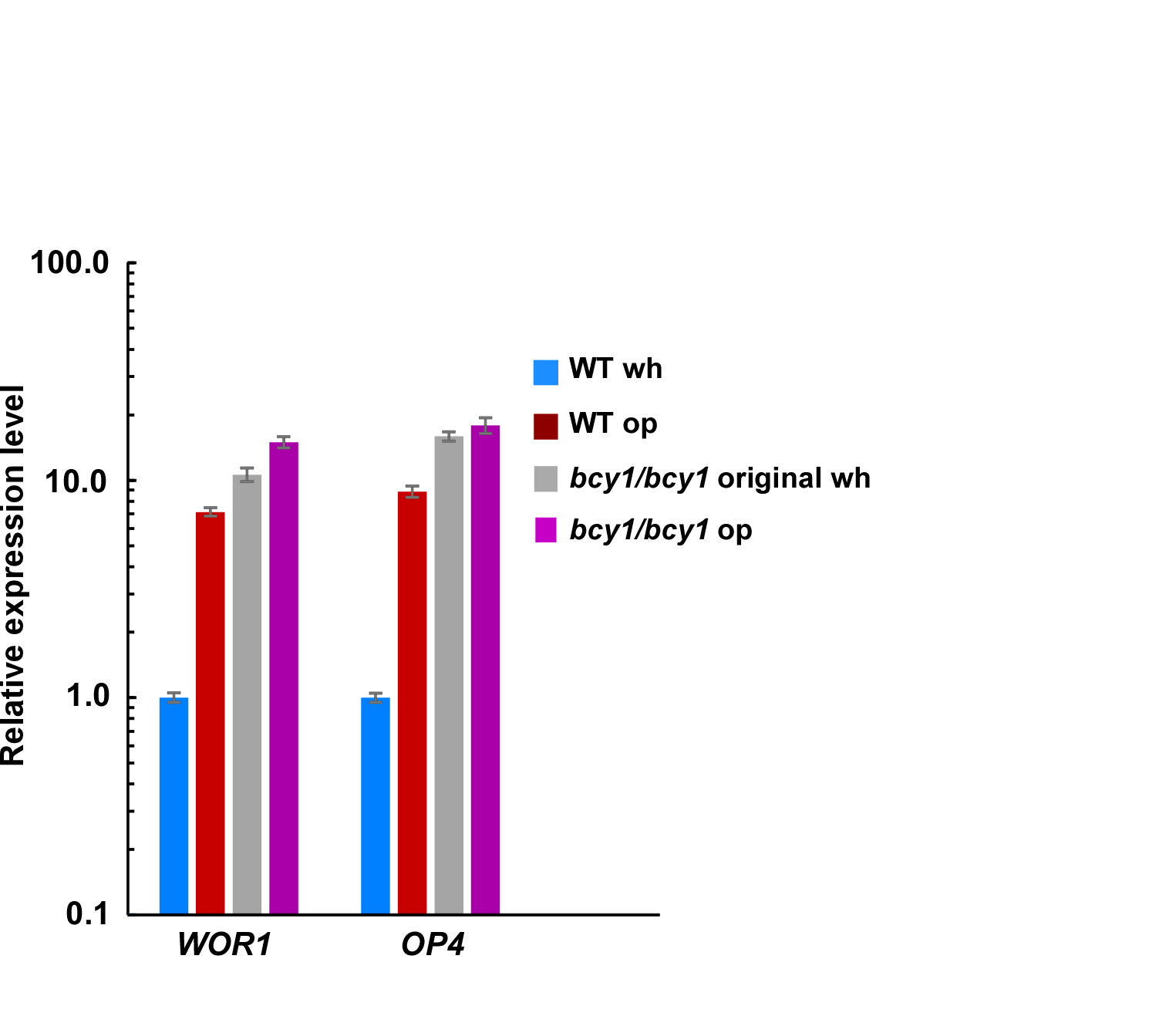

Supplement: Figure S1 — Relative gene expression levels in white and opaque cells. Cells collected from Lee's GlcNAc medium plates (three days, at 25°C) were used for qRT-PCR assays. The values of the expression level of each gene in white cells of the WT strain were set as “1.” White and opaque cells of the WT served as controls. bcy1/bcy1 op, opaque cells plated on Lee's GlcNAc medium; bcy1/bcy1 original wh, white cells plated on Lee's GlcNAc medium. ACT1 served as the reference gene for normalization. [file Image1.TIF]
