# Supplementary material for: The Regulatory Subunit of Protein Kinase A (Bcy1) in Candida albicans Plays Critical Roles in Filamentation and White-Opaque Switching but Is Not Essential for Cell Growth
Source: Front Microbiol. 2017 Jan 5;7:2127. doi: 10.3389/fmicb.2016.02127 (PMC5215307; doi:10.3389/fmicb.2016.02127)
Supplement: Table S1 — Strains used in this study. [file Table1.DOCX]

**Table S1. Strains used in this study**

| Strain name | Parent strain | Genotype | Reference |
| --- | --- | --- | --- |
| SN152 | SC5314 | MTL**a**/α arg4Δ/arg4, leu2Δ/leu2, his1Δ/his1,URA3/ura3*::λimm434*, IRO1/iro1*::λimm434*, | 1 |
| WT  (GH1350) | SN152 | MTL**a**/α arg4Δ/ARG4, leu2Δ/LEU2, his1Δ/HIS1,arg4Δ/arg4, leu2Δ/leu2, his1Δ/his1,URA3/ura3*::λimm434*, IRO1/iro1*::λimm434*, | This study |
| *bcy1/BCY1* | SN152 | MTL**a**/α *bcy1:: HIS1/BCY1 leu2/leu2 ura3Δ/URA3 his1 /his1 arg4/arg4 iro1/IRO1* | This study |
| *bcy1/bcy1* | *bcy1/BCY1* | MTL**a**/α *bcy1:: HIS1L / bcy1:: LEU2*  *leu2/leu2 ura3Δ/URA3 his1 /his1*  *arg4/arg4 iro1/IRO1* | This study |
| *bcy1/bcy1+ARG4* | *bcy1/bcy1* | MTL**a**/α *bcy1:: HIS1L / bcy1:: LEU2 /arg4::ARG4 leu2/leu2 ura3Δ/URA3 his1 /his1 arg4/arg4 iro1/IRO1* | This study |
| *bcy1/bcy1+BCY1* | *bcy1/bcy1* | MTL**a**/α *bcy1:: HIS1L / bcy1:: LEU2/ BCY1p-BCY1-ARG4 leu2/leu2 ura3Δ/URA3 his1 /his1arg4/arg4 iro1/IRO1* | This study |
| SN152 **a** | SN152 a/alpha | As SN152 a/alpha*,* but *MTL*α/*mtl***a**::*FRT-SAT1 -FRT* | This study |
| *bcy1/bcy1* **a** | SN152 **a** | As SN152 **a**, *bcy1:: HIS1L / bcy1:: LEU2*  *leu2/leu2 ura3Δ/URA3 his1 /his1*  *arg4/arg4 iro1/IRO1* | This study |
| WT+pACT1 | WT | As WT, but ADE2/ade2::ACT1p-URA3 | This study |
| WT+ACT1p*-TPK1* | WT | As WT, but ADE2/ade2::ACT1p –TPK1-URA3 | This study |
| WT+ACT1p*-TPK2* | WT | As WT, but ADE2/ade2::ACT1p –TPK2-URA3 | This study |
| *bcy1/bcy1*+pACT1 | *bcy1/bcy1* | As *bcy1/bcy1*, but ADE2/ade2::ACT1p -URA3 | This study |
| *bcy1/bcy1*+pACT1*-TPK1* | *bcy1/bcy1* | As *bcy1/bcy1*, but ADE2/ade2::ACT1p –TPK1-URA3 | This study |
| *bcy1/bcy1*+pACT1*-TPK2* | *bcy1/bcy1* | As *bcy1/bcy1*, but ADE2/ade2::ACT1p –TPK2-URA3 | This study |

**References**

1 Noble SM, Johnson AD. Strains and strategies for large-scale gene deletion studies of the diploid human fungal pathogen Candida albicans. Eukaryot Cell. 2005;4(2):298-309.
