# Supplementary material for: The Regulatory Subunit of Protein Kinase A (Bcy1) in Candida albicans Plays Critical Roles in Filamentation and White-Opaque Switching but Is Not Essential for Cell Growth
Source: Front Microbiol. 2017 Jan 5;7:2127. doi: 10.3389/fmicb.2016.02127 (PMC5215307; doi:10.3389/fmicb.2016.02127)
Supplement: Table S2 — Primers used in this study. [file Table2.DOCX]

**Table S2. Primers used in this study**

| **Name** | **Sequence (5’ to 3’)** | **Purpose** |
| --- | --- | --- |
| Marker-F | CCGCTGCTAGGCGCGCCGTGACCAGTGTGATGGATATCTGC | Amplification for *ARG4, HIS1, LEU2, URA3* fragments used in fusion PCR |
| Marker-R | GCAGGGATGCGGCCGCTGACAGCTCGGATCCACTAGTAACG |  |
| BCY1-orf-F | TTCTAGTGGTGTGAGCATTG | *BCY1* deletion confirmation |
| BCY1-orf-R | TTTTCTTAGAGTTTGCGTCC |  |
| FP-BCY1-5F | TTTGTTGTTATCACGTGAAGTGTCG | For *BCY1* knockout used in fusion PCR |
| FP-BCY1-5R | CACGGCGCGCCTAGCAGCGGCTCACTCCCAAAGTAATTTCTCCTT |  |
| FP-BCY1-3F | GTCAGCGGCCGCATCCCTGCCTTGTTGTCAGGTATATACGAGATG |  |
| FP-BCY1-3R | GAAAGTGAGAACCTCTTGTAGTGTT |  |
| BCY1-CH-F | ATTAATTGTGCTTACGTGGC | *BCY1* deletion confirmation |
| BCY1-CH-R | AAAATGCTTTGGAACCTTGG |  |
| BCY1-5F-COM | ATGGTCATCAATTGTTCATTC | For *BCY1* complementation used in fusion PCR |
| BCY1-5R-COM | CACGGCGCGCCTAGCAGCGGGCTGCCATCTCGTATATACCTG |  |
| BCY1-3F-COM | GTCAGCGGCCGCATCCCTGCCCTTTTGCGAAACATGGTAA |  |
| BCY1-3R-COM | GAAAGTGAGAACCTCTTGTAGTGTT |  |
| Marker-LEU2-F CH | AGAATTCCCAACTTTGTCTG | *BCY1* deletion marker confirmation |
| Marker-LEU2-R CH | \| AAACTTTGAACCCGGCTGCG \| \| --- \| |  |
| Marker-HIS1-F CH | ATTAGATACGTTGGTGGTTC |  |
| Marker-HIS1-R CH | \| AACACAACTGCACAATCTGG \| \| --- \| |  |
| OE-TPK1 F | ATTATAGATATCACATCCATGGAACCAGCAG | Overexpression plasmid pACT1-TPK1-URA3 |
| OE-TPK1 R | ATTAATAAGCTTTAGAACTACCAAATCATCCC |  |
| OE-TPK2 F | ATTATAAGGCCTGACAATCATCAACAACAACAGC | Overexpression plasmid pACT1-TPK1-URA3 |
| OE-TPK2 R | ATTAATAAGCTTACCTCGATCAGATAAAGAATGTCC |  |
